# Barriers and Facilitators of Online Patient Portals to Personal Health Records Among Persons Living With HIV: Formative Research

**DOI:** 10.2196/resprot.2302

**Published:** 2013-01-22

**Authors:** Amneris E Luque, Adjuah van Keken, Paul Winters, Michael C Keefer, Mechelle Sanders, Kevin Fiscella

**Affiliations:** ^1^Department of Medicine, Infectious Disease DivisionUniversity of Rochester School of Medicine & DentistryRochester, NYUnited States; ^2^Center for Community HealthUniversity of RochesterRochester, NYUnited States; ^3^Department of Family MedicineHighland HospitalUniversity of Rochester Medical CenterRochester, NYUnited States; ^4^Developmental Center for AIDS ResearchUniversity of Rochester School of Medicine & DentistryRochester, NYUnited States

**Keywords:** HIV, health records, personal medical informatics, education of patients

## Abstract

**Background:**

Federal meaningful use standards are promoting adoption of online portals to personal health records (PHRs). However, relatively little is known regarding barriers and facilitators for vulnerable groups such as persons living with human immunodeficiency virus (PLWH).

**Objective:**

The objective of this study was to assess barriers and facilitators to use of online PHRs among PLWH.

**Methods:**

We conducted formative research using a written waiting room survey among 120 PLWH regarding barriers and facilitators of portal PHR use. We supplemented findings with data collected from a PLWH focus group, where some members had personal experience with use of a portal.

**Results:**

The survey had 90 respondents. Eight PLWH participated in the focus group. Most patients (77/90, 86%) reported having at least some experience using the Internet and most expressed interest in features offered by the portal. Notably, 70% (63/90) expressed some interest in being taught how to use it to communicate with their provider. Focus group themes reinforced these findings, but also voiced concern regarding access to private computers.

**Conclusions:**

Many PLWH in our sample have experience using computers and most are interested in PHR features. However, computer or broadband access and privacy are important barriers.

## Introduction

Personal health records (PHRs), typically accessible through secure Web-based portals, represent a practical way for patients to access their health information anytime and anywhere [1]. The federal meaningful use standards require providers to make PHRs accessible to patients [2]. Some predict that patient portals will eventually become the primary means through which patients access their PHRs, request and cancel physician appointments, request medication refills, view test results, and exchange messages with their health care team [1]. However, the emergence of online PHRs may worsen the disparity of health care access on the basis of the well-documented digital divide [3,4].

Mounting data point to the growing sociodemographic disparities in patients’ use of Web-based portals for PHRs [5-8]. This disparity in access is particularly relevant for patients living with chronic conditions such as human immunodeficiency virus (HIV) who are a racial/ethnic minority and disproportionately poor [9]. Data from early adopters of PHRs suggest that theses disparities in PHR use will emerge among persons living with HIV (PLWH) [10].

Barriers to Web-enabled technology can be largely grouped into 3 categories, including cost, knowledge and attitude, and skills [11]. Cost is the most obvious contributor. Low-income families often report they are not able to afford the cost of personal computers and monthly fees for broadband access [11]. However, some have access to computers or the Web through friends, family, work, libraries, or handheld devices [11]. The second contributor relates to attitude. Many nonusers report they see little value in computers and/or the Internet [11]. This is particularly true of older persons who grew up in an era without this technology in social networks that reinforces these negative attitudes through social norms. Lastly, computer use and attitudes are shaped by self-efficacy and skill level related to computer and Internet use. Many persons with low education and older persons lack the necessary skills and confidence to access health information online [11,12]. Interestingly, neither mental health nor substance use disorders is correlated to portal use by PLWH [13].

We conducted formative research to understand the barriers and facilitators on PLWH in using key features associated with PHRs. To obtain a more complete picture, we supplemented the survey with PLWH with input from a PLWH focus group, where members had varied experience with online PHR.

## Methods

### Survey

Over a 2 week period, 120 written surveys available in English and Spanish were distributed to patients 18 years and older by the front staff during their appointments at an HIV clinic that serves roughly 1000 HIV patients. Survey questions were adapted from the Health Information National Trends Survey (HINTS) and addressed barriers and facilitators of Web use and the level of interest to key PHR features, such as scheduling appointments, requesting refills, viewing test results, and exchanging electronic messages with one’s providers [14]. The final survey contained 18 items, including patient demographic characteristics (age, sex, education, insurance, and marital status), computer access and comfort, level of interest in specific features available through patient portals, and desire for assistance. Over a 2-week period in November 2011, the survey was handed out to all patients 18 years of age or older that checked-in for an appointment.

### Focus Group

**Figure 1:**
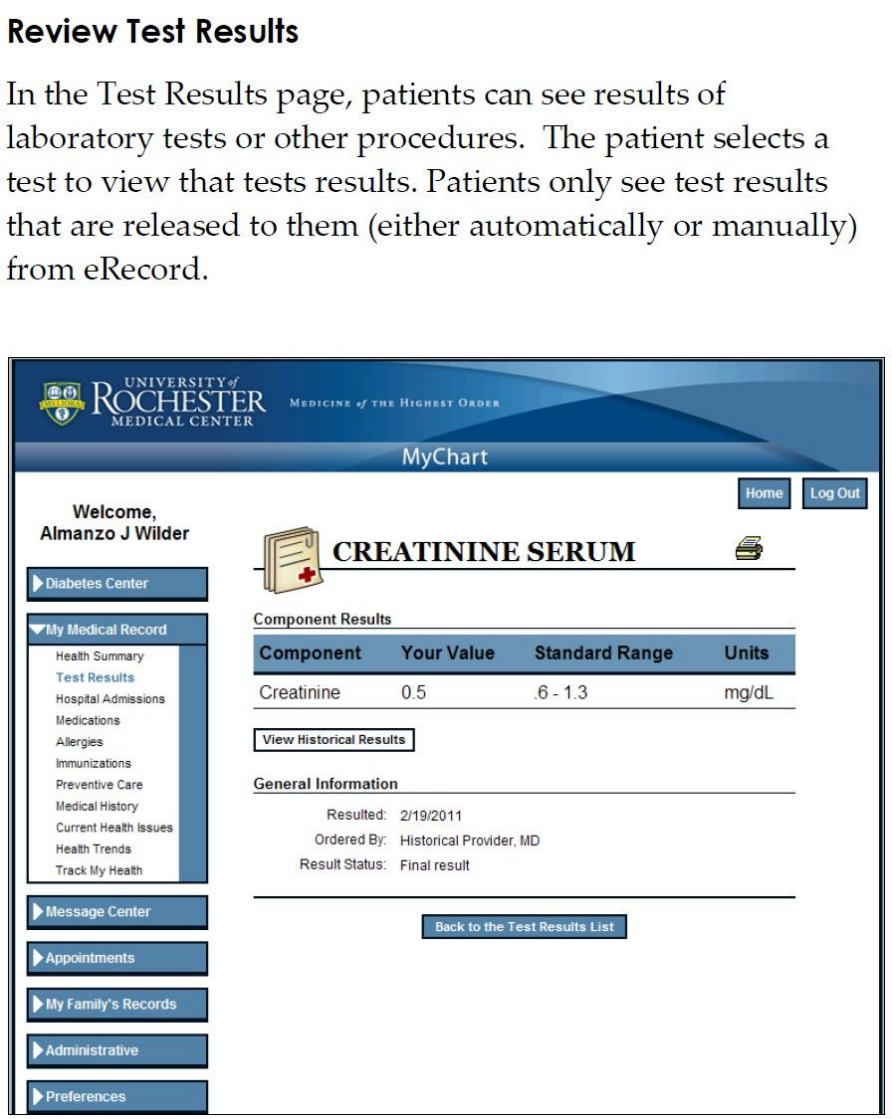
Test results in personal health records.

**Figure 2:**
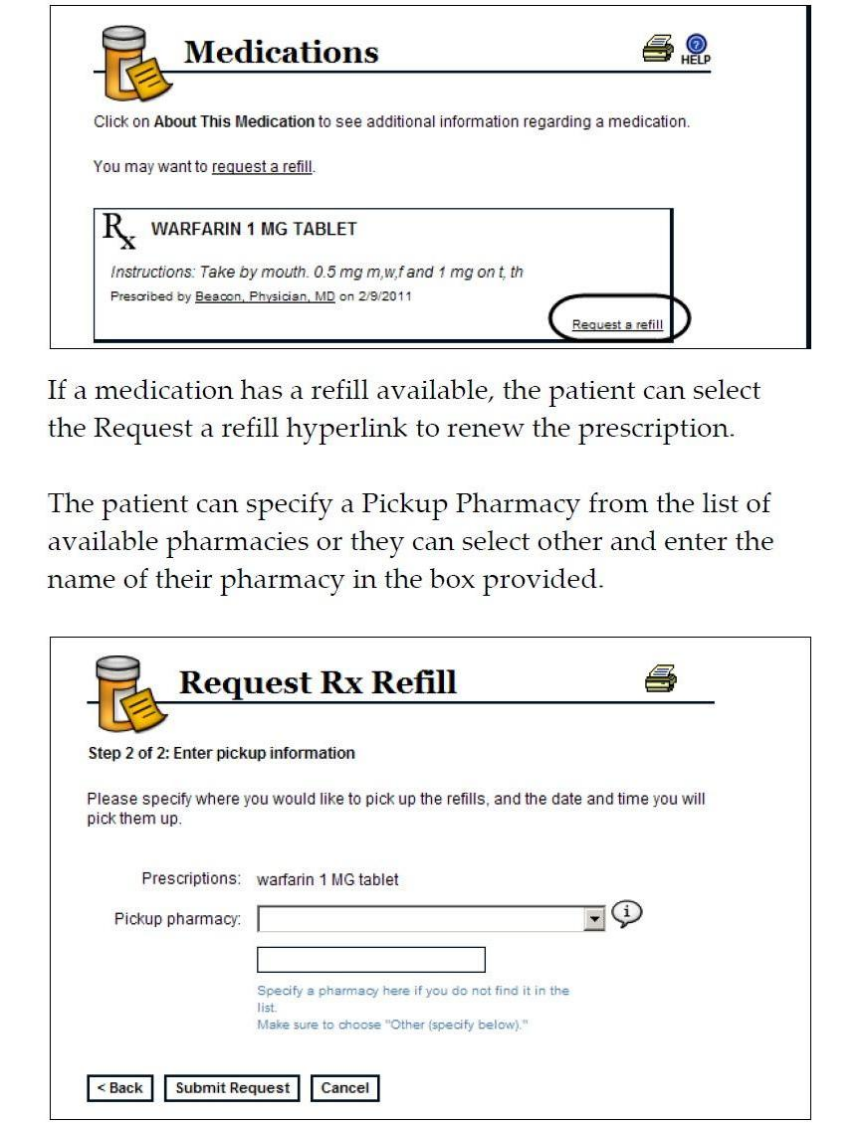
Prescription refill requests in personal health record.

An online PHR focus group was formed by 8 PLWH participants recruited by offices and organizations that provided care for PLWH. A trained research assistant conducted the group, which lasted for one hour. All participants provided verbal informed consent and were compensated for their time and travel. The research assistant began with a brief presentation of Web-enabled PHRs using slides that included screenshots of a PHR. (Figures 1 and 2). The presentation was followed by a series of open-ended questions regarding barriers and facilitators of PHR use among PLWH. The research assistant concluded the group session by inviting participants to offer suggestions to improve access. Data were compiled based on an audio recording of the focus group and notes by the research assistant. The survey and focus groups were approved the University of Rochester Institutional Review board.

### Data Analysis

#### Survey Analysis

We examined univariate responses for each item and collapsed categories to create dichotomous categories. We compared responses across items using chi-square statistics. We examined independent associations among multiple factors using logistic regression.

#### Focus Group Analysis

The principal investigator KF and the research assistant analyzed the focus group using qualitative methods. We assigned participant responses to de novo categories and then developed codes for each category. These codes included specific barriers to use of PHR portals (lack of physical access, privacy concerns, computer literacy, and patient interest) and facilitators (pro-active engagement, easy-to-use technology, training, and privacy). The codes were then applied to the entire data for analysis [15].

## Results

### Survey Findings

Of the 90 patients (Table 1), most identified themselves as black (38/90, 43%) or 8% (7/90) Hispanic, male (54/90, 61%), 64% had a high school education or less and 38% indicated they had Medicaid as their primary insurance coverage, with 22% having private insurance and 20% relying on the AIDS Drug Assistance Program (ADAP). Thus, while we did not ask about income, based on educational levels and insurance, the sample is likely low-income.

**Table 1:** Demographic characteristics.

Patient characteristic (N=90)		n (%)
**Age**		
	< 35	24 (29)
	35-49	33 (39)
	> 50	27 (32)
**Sex**		
	Female	32 (36)
	Male	54 (61)
	Transgendered	3 (3)
**Race/Ethnicity**		
	Black	38 (43)
	Hispanic/Latino	7 (8)
	White	39 (44)
	Other	5 (6)
**Education**		
	< high school	27 (30)
	high school	31 (34)
	> high school	36 (36)
**Marital Status**		
	Single	57 (63)
	Married	16 (18)
	Divorced/separated	14 (16)
	Widowed	3 (3)
**Health Insurance**		
	Medicaid	32 (39)
	Private	18 (22)
	ADAP	17 (20)
	Other	15 (18)
	None	1 (1)

Surprisingly, most respondents reported at least monthly Internet use despite only about half owning a computer (Table 2). Other means for accessing the Internet access included a smartphone, work, friend, family, or library computer. Barriers cited by respondents were cost (16/90**,** 18%), lack of interest (6/90, 22%) and do not know how to use (5/90, 19%).

**Table 2:** Internet use and barriers to use.

Internet use and barriers (N=90)		n (%)
**Monthly or more Internet use**		
		77 (82)
**Primary location of Internet use**		
	Own computer	45 (52)
	Smartphone	7 (8)
	Work computer	12 (14)
	Friend/Family computer	15 (17)
	Library computer	2 (2)
	Other	5 (6)
**Barriers to use**		
	Do not know how to use	5 (19)
	Costs too much to use	16 (18)
	Not interested in using	6 (22)

Most respondents reported at least some interest in obtaining test results and scheduling appointments online (Table 3). In addition, most (77/90, 86%) reported they would use the computer if available on-site at the clinic and most (54/90, 70%) expressed an interest in being taught how to use a patient portal to communicate with their provider. In multivariate analysis, no single factor was statistically associated with interest in PHRs suggesting that interest was distributed across all groups fairly equally. However, participants who reported more interest in PHR features were significantly more likely to report interest in being taught how to communicate with their providers (**P*<.*001).

**Table 3:** Interest in patient portal features and assistance.

Features (N=90)		n (%)
**Interest in key online PHR features**		
	Obtaining test results online	76 (86)
	Schedule appointments online	63 (73)
	Refilling prescriptions online	74 (83)
**Interest in improve access or assistance**		
	Use a computer in waiting room	77 (86)
	Having someone teach you how to use it to communicate with your doctor	54 (70)

### Focus Group Findings

The focus group participants cited the lack of Internet access and not knowing how to use these online PHRs as barriers, but mentioned an additional barrier to use of online portals—privacy when accessing a portal outside of one’s home. Most of the participants reported they did not have home Web access. While the group acknowledged the availability of Web access through libraries and homes of friends and family, most were concerned about using a public computer or a computer in someone else’s home to access PHR. One participant commented, “You wouldn’t try to access your online bank account in public. Why would you access your personal health record there?” When asked about use of computers within clinics, participants preferred use of a small hand held device such as an iPad to that of a desktop computer because they felt it would be easier to preserve privacy by concealing personal information on the screen and also easier to learn to use. When the issue of computer literacy was raised, participants agreed that this represented a barrier but did not view it as insurmountable. Many felt that it would be feasible to train patients in 10-15 minutes to use a touch screen device such an iPad on-site at clinics.

## Discussion

PLWH in our sample reported notable interest in use of Web-enabled PHRs. This finding is consistent with findings from national surveys that document significant interest in PHRs [16,17]. It bodes well for greater engagement of PLWH in their self-management and is a means for potentially improving medication adherence through greater self-monitoring of test results, efficient medication refills, and greater access to providers through electronic messaging. Yet, promoting adoption of PHRs will require addressing key barriers including knowledge, attitudes, access, cost, skills, and self-efficacy. These barriers identified in our survey are broadly similar to those from the national survey, which included lack of perceived need, costs, time required, and lack of interest in computers [18]. In addition, patients in the national surveys cited concerns with privacy [18]. This concern also emerged from the focus group in the context of using a public computer.

Ensuring that unequal adoption of Web-based PHRs does not worsen existing disparities requires practical strategies to address incentives and various barriers [19]. The first barrier identified by our participants was physical access, which is driven in part by the cost of computers and monthly broadband access fees. This might be mitigated if public libraries and medical providers offered on-site access to Web-enabled devices. However, it means offering this access in ways that protect the privacy of those accessing their PHR while they are viewing their health information.

Second, the barriers of computer skills and self-efficacy need to be resolved [20]. Most patients expressed interest in on-site guidance and instruction. Given relatively low rates of use of PHRs (when available) including by PLWH and emerging disparities [5-8,21-23], pro-active engagement of patients with limited computer literacy coupled to basic hands-on instruction may be needed to forestall these disparities [24]. Personal demonstration and role modeling use of the technology may spark interest among those with limited knowledge about PHRs and among those who see limited benefit. Our findings suggest that patients will welcome on-site availability and instruction provided that their privacy is ensured.

These findings are limited by our methods. Our sample was based on recruitment from a waiting room. This precludes assessment of response bias. Thus, it is possible that patients with the lowest computer literacy might have been less likely to respond, perhaps viewing the survey as less relevant to them. Similarly, we recruited patients from a single practice. Although the demographic characteristics of the responders are similar to that of PLWH nationally we cannot be sure that the findings generalize to other settings. Last, we conducted only one focus group. It is possible that additional themes would emerge with additional groups.

In conclusion these findings provide cause for some cautious optimism. They suggest that PLWH are interested in features offered by PHR, but that significant barriers remain. Some of these barriers can potentially be overcome through on-site online PHR access coupled to training.

## Abbreviations

ADAP: AIDS drug assistance program
HINTS: Health Information National Trends Survey
HIV: human immunodeficiency virus
PHR: personal health record
PLWH: person living with HIV
